# Comparison of Protective Potency of DNA and Live Vaccines Expressing A2-CPA-CPB^-CTE^ Antigens against Visceral Leishmaniasis in Syrian Hamster as Preliminary Study

**DOI:** 10.18502/ijpa.v15i3.4203

**Published:** 2020

**Authors:** Yasaman TASLIMI, Farnaz ZAHEDIFARD, Tahereh TAHERI, Delaram DOROUD, Sakineh LATIF DIZAJI, Noushin SALJOUGHIAN, Sima RAFATI

**Affiliations:** 1. Department of Immunotherapy and Leishmania Vaccine Research, Pasteur Institute of Tehran, Tehran, Iran; 2. Quality Control Department, Production and Research Complex, Pasteur Institute of Tehran, Tehran, Iran; 3. Department of Laboratory Animal Science, Pasteur Institute of Tehran, Tehran, Iran

**Keywords:** Visceral leishmaniasis, DNA vaccination, Live vaccination, Hamster immunization

## Abstract

**Background:**

Visceral leishmaniasis is the most severe form of leishmaniasis caused by *Leishmania* (*L.*) *donovani* complex. Drug-resistant strains have been developed as a consequence of the current chemotherapeutic interventions, which has increased the need for advanced preventive and therapeutic strategies. A2-CPA-CPB^-CTE^-recombinant strain of *L. tarentolae*, which is non-pathogenic to humans, was shown protective in live vaccine as well as its DNA vaccine counterpart in both murine and canine models.

**Methods:**

We evaluated the effectiveness of these DNA and live vaccination harboring A2-CPA-CPB^-CTE^ in protecting hamsters against *L. infantum* infection using prime-boost regimens, namely DNA/DNA and Live/Live (n=9 hamsters per group). Cationic solid lipid nanoparticles (cSLN) were utilized as an adjuvant for DNA priming and electroporation for boosting DNA. At different time points post-challenge, parasite burden and body weight as well as humoral immune responses were measured.

**Results:**

Both immunization strategies partially protect hamsters against *L. infantum* challenge. This protective immunity is associated with remarkable decrease in parasite load in liver and spleen of vaccinated hamsters eight weeks after challenge compared to control group.

**Conclusion:**

Both test groups (DNA/DNA and Live/Live) elicited high levels of IgG2 and total IgG as humoral immune responses and lower level of parasite propagation in both liver and spleen.

## Introduction

Leishmaniasis is an infectious disease with different clinical spectra including the severe form of visceral leishmaniasis (VL) with an annual incidence of approximately 50,000–90,000 new cases (1). The disease is prevalent in Latin America, the Mediterranean Basin and Asia, causing serious public health problem. If left untreated, the mortality rate in developing countries can be as high as 95% within two years after the infection (1).

VL is endemic in different provinces of Iran and dogs, jackals and foxes are the main reservoirs (2, 3). In addition, emergence of worldwide drug-resistant forms have forced researcher to find new solutions (4).

Despite the extensive knowledge about the immunology of this disease from experimental models and parasite biology, there is no available vaccine against any form of leishmaniasis for human use (5, 6). First generation vaccine against leishmaniasis has been evaluated on dog model (7, 8). In a study, gentamicin attenuated *L. infantum* parasite were tested against *L. infantum* infection in dogs. Vaccinated dogs had elevated levels of CD4^+^ and CD8^+^ T cells (9). Another live attenuated *L. donovani* species lacking a growth regulating gene (*Ldcen 1*−*/*−) were tested against *L. infantum* infected dogs resulted to Th1 response production in test group which can limit infection more easily (10). Second generation vaccines consist of subunit proteins or crude antigens of whole parasites. In an experiment in Brazil, dogs were vaccinated with *L. braziliensis* promastigote protein and saponin showed decrease level of parasite burden and elevated levels of IFN-γ, also increased level of total IgG and subclasses (IgG1 and IgG2) were observed (11).

Maintaining a limited pool of live parasites after recovery from the infection is essential for long term immunity (12). This can be met by using live attenuated vaccines or nonpathogenic *Leishmania* species (13). The protozoan parasite *L. tarentolae*, so far known to naturally infect only lizards, has never been associated with any form of leishmaniasis and is therefore considered as non-pathogenic (14). Using this species as a carrier, different antigens have been examined for their potential capability to induce protective immunity. A2 gene family in *L. donovani* was the first gene family identified to be expressed only in the amastigote stage. A2 is shown to play a deterministic role in the visceralization process, and its recombinant protein is highly immunogenic in mice (1, 15). Importantly, A2 antigen is not present in *L. tarentolae,* and administration of live recombinant *L. tarentolae* parasites stably expressing A2 can increase the level of IFN-γ production and consequently protect BALB/c mice against *L. infantum* challenge (16). In addition, Cathepsin L-like Cysteine proteinases (CPs) have generated significant interest as protective antigens. Among others, type I and II CPs have been shown to induce protective immunity in heterologous regimes as prime-boost vaccination (17–21).

Furthermore, DNA vaccination offers an interesting substitute to conventional non-replicating vaccines. Intracellular production of antigens from delivered DNAs can result in both humoral and cellular immune responses (22). Cocktail DNA vaccines have been examined and it was shown that the combination of cysteine proteinase CPA/CPB and CPA/CPB^CTE^ could induce stronger protection against cutaneous and visceral leishmaniasis than the individual forms do (20, 21, 23, 24). However, regardless of many years of research, safety and efficient delivery of pDNA to initiate appropriate immune responses remains one of the major obstacles in bringing DNA vaccination into clinical trials (25). Therefore, there is still an urgent need for improvement, safety and efficient adjuvants and/or delivery systems in order to increase the immunogenicity of the current DNA vaccine candidates (25). Electroporation is one of the methods to facilitate the delivery of DNA vaccines into skin or skeletal muscle (26).

Electroporation has stand out as a method for gene delivery due to its site-specific nature and high efficacy (27). It is important to note that, cationic solid lipid nanoparticles (cSLN) serves as an advanced delivery technology with possibility for further formulation to overcome cost, toxicity, and shelf-life stability issues (28). These nanoparticles consist of physiologically well-tolerated ingredients mostly approved for pharmaceutical application in humans (29).

Herein, the tri-fusion of A2-CPA-CPB^-CTE^ genes were utilized as a DNA vaccine either formulated with cSLN or delivered via electroporation along with a recombinant *L. tarentolae* expressing the tri-fusion gene as a live vaccination strategy against visceral leishmaniasis in the best modalities resulted from our previous studies in mice. Mouse and hamsters develop a slow chronic infection that can be kept under control for variable time period. Hamsters appear to be more appropriate hosts and future development of immunological tools would greatly enhance the value of this model. Accordingly DNA cSLN/DNA electroporation and Live/Live were examined in hamsters as an animal model that mimics the natural course of VL infection.

## Materials and Methods

### Ethical approval

All hamster experiments including maintenance, animals handling and blood collection procedures were approved by Institutional Animal Care and Research Advisory Committee of Pasteur Institute of Iran (Education Office), based on the Specific National Ethical Guidelines for Biomedical Research issued by the Research and Technology Deputy of Ministry of Health and Medicinal Education of Iran (issued in 2005).

### Parasite

A recombinant strain of *L. tarentolae* parasites expressing A2 and CP genes was already available from our previous study (30). The *L. tarentolae* and recombinant *L. tarentolae* A2-CPA-CPB^-CTE^ parasites were grown in M199 (Sigma, Missouri, USA) supplemented with 5% heat-inactivated fetal calf serum (FCS, Gibco) and 0.1 mM adenosine, 40 mM HEPES (pH 7.2), 5 μg/mL hemin (all chemicals purchased from Sigma), and 50 μg/mL gentamicin (Biosera, France) at 26 °C. The *L. infantum* strain MCAN/ES/98/LLM-877 was kindly provided by WHO collaborating center for leishmaniasis, Servicio de Parasitología, Centro Nacional de Microbiología, Instituto de Salud Carlos III, Madrid, Spain and kept in virulent state by continuous passage in hamsters. Amastigotes were isolated from the previously-infected hamsters’ spleens and cultured in Novy-MacNeal-Nicolle (NNN) media and prepared for the infectious challenge as described previously (30). Parasites were frozen and thawed (F/T) and protein concentration of the lysate was determined by bicinchoninic acid reagent (BCA, PIERCE, Chemical Co. Rochford III).

### Immunization schedules and infectious challenge

Adult male hamsters (3–4 month old, 100–120 gram) were obtained from the breeding stock maintained at the Pasteur Institute of Iran. Immunization experiments were carried out in three groups of hamsters (n = 9); group 1 (G1: DNA cSLN/DNA electroporation 50 μg of pcDNA-A2-CPA-CPB^-CTE^), group 2 (G2: Live/Live 3×10^7^ *L. tarentolae*A2-CPACPB^-CTE^), group 3 (G3: PBS control) (Table 1). All groups were immunized via subcutaneous route. Booster immunization was performed three weeks after the first immunization.

**Table 1:** Vaccine modalities and routes of inoculation in different groups

***Groups***	***Priming Subcutaneous route (day 0)***	***Booster Subcutaneous route (day 21)***	***Challenge Intracardiac route (day 42)***	***Vaccine Modality***
Group 1	pcDNA-A2-CPA-CPB^-CTE^-SLN 50 ug/100ul	pcDNA-A2-CPA-CPB^-CTE^ electroporation 50 ug/100ul	*L. infantum* 10^7^/100ul	DNA/DNA
Group 2	*L. tarentolae* (A2-CPA-CPB^-CTE^) 3×10^7^/100ul	*L. tarentolae* (A2-CPA-CPB^-CTE^) 3×10^7^/100ul	*L. infantum* 10^7^/100ul	Live/Live
Group 3	PBS/100ul	PBS/100ul	*L. infantum* 10^7^/100ul	Control

**Fig. 1:**
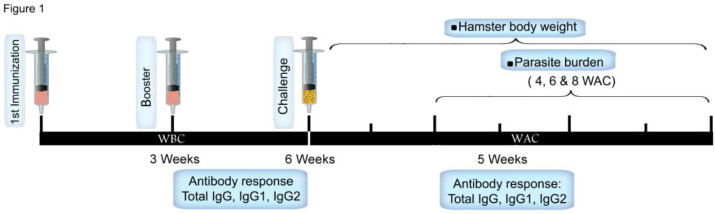
Schematic presentation of vaccination and infectious challenge in hamster**.** The animals were immunized twice with three weeks intervals and challenged with *L. infantum* three weeks after booster immunization. Antibody response and parasite burden were assessed at different periods. WBC: weeks before challenge, WAC: weeks after challenge

For infectious challenge, three weeks after booster immunization all hamsters were challenged with 10^7^ stationary phase *L. infantum* promastigotes by intracardial route (Fig. 1).

### Determination of Antigen-specific antibody responses in hamsters

Hamsters’ sera samples were measured by ELISA for specific antibodies response including Total IgG, IgG1 and IgG2 against either rA2, rCPs or *Leishmania* F/T before and five weeks after challenge. Concisely, 10 μg/ml of rA2, rCPA, rCPB and *L. tarentolae-*A2-CPA-CPB^-CTE^ or *L. infantum* F/T were coated in 96-well plates (Greiner) and incubated overnight at 4 °C. Plates were blocked with 100 μl of 1% BSA in PBS at 37 °C for 2 h to prevent specific binding. Sera were added (with dilution of 1:100) and plates were incubated 2 h at 37 °C. After three washes, biotin mouse anti-Armenian and Syrian hamster IgG1 (1:2000, BD Bioscience), anti-Golden Syrian hamster IgG (1:20000, Rockland) or biotin mouse anti-Syrian hamster IgG2 (1:2000, BD Bioscience) were added and incubated for 2 h at 37 °C. Plates were washed and incubated for 30 min at 37 °C with Peroxidase Substrate System (KPL, ABTS). Reactions were stopped by addition of 1% SDS and the optical absorbance was measured at 405 nm. For biotinylated antibodies there is an extra step before addition of substrate by adding streptavidin, and then the plate was covered and incubated for 20 min at room temperature.

### Determination of parasite burden in hamsters by Serial Dilution Assay

Three hamsters from each group were sacrificed at 4, 6 and 8 WAC and parasite burdens were determined. Small pieces of spleen and liver were taken in sterile condition, weighed and homogenized with a tissue grinder in 2 ml of Schneider’s *Drosophila* medium supplemented with 20% FCS and gentamicin (0.1%), and then serial dilutions ranging from 1 to 10^−20^ were prepared and incubated at 26 °C. The presence of motile promastigotes was examined at two time points of 7 and 14 d and the number of parasites per gram of hamster’s tissue was determined using the following formula: parasite burden = −log_10_ (tissue weight/parasite dilution) (31, 32).

### Statistical analysis

Statistical analysis was performed using Graph Pad Prism 5.0 for Windows (Graph pad Software Inc. 2007, San Diego, Calif., USA). All the data were analyzed with oneway ANOVA (Multiple-comparison Tukey post Hoc test) and Student’s *t*-test. A *P-*value of less than 0.05 was considered significant.

## Results

### Immunization with both DNA and live recombinant L. tarentolae of A2-CPACPB-^CTE^tri-gene fusion protects hamsters against L. infantum infectious challenge

**Fig. 2:**
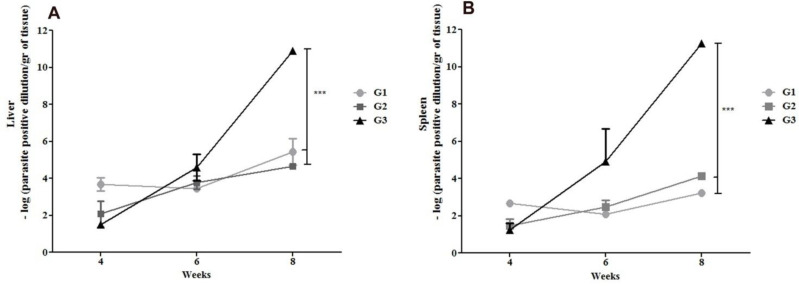
Liver and spleen parasite loads in vaccinated and control hamster groups following infectious challenge with *L. infantum*. The parasite load in the liver (A) or spleen (B) was measured by Serial Dilution Assay at 4, 6 and 8 WAC. Parasite load in liver (A) and spleen (B) between groups G1 to G3 were compared (3 hamsters per group per time point). Results are shown as mean±SD. G1 [vaccinated with DNA-A2-CPA-CPB^-CTE^cSLN (prime) and DNA-A2-CPA-CPB^-CTE^ (boost)]; G2 [vaccinated with Live *L. tarentolae*-A2-CPACPB^-CTE^ (prime) and Live *L. tarentolae*-A2-CPA-CPB^-CTE^ (boost)]; and G3 (control PBS). Student’s *t* test and ANOVA tests were used for statistical analysis (***P<0.001)

Immunization experiments were carried out in three groups of hamsters (n = 9); as explained before. Three hamsters from each group were sacrificed at 4, 6 and 8 WAC and parasite burdens were determined via Serial Dilution Assay. Two months post infection we observed significantly lower level of parasite load in liver and spleen of hamsters immunized with DNA-A2-CPA-CPB^-CTE^-cSLN (prime) and DNA-A2-CPA-CPB^-CTE^ via electroporation (boost) (G1) and Live *L. tarentolae-*A2-CPA-CPB^-CTE^ (prime and boost) (G2) compared with control group G3 (Fig. 2A and B).

Besides, it is observed that parasite burden increases more rapidly from week 4 till 8 (WAC) in control group in comparison with vaccinated groups both in liver and spleen (Fig. 2A and B). In liver, parasite burden slopes of control group G3 is approximately 5 folds higher than vaccinated G1 and 4 folds higher than vaccinated G2. In spleen, parasite burden slopes of control group G3 is approximately 16 folds higher than vaccinated G1 and 4 folds higher than vaccinated G2. Therefore, we can conclude that parasite burden has been controlled in both vaccinated groups G1 and G2 in comparison to control group G3.

### Immunization with DNA vaccination and live recombinant L. tarentolae-A2-CPACPB^-CTE^ stimulate the IgG2 production

**Fig. 3:**
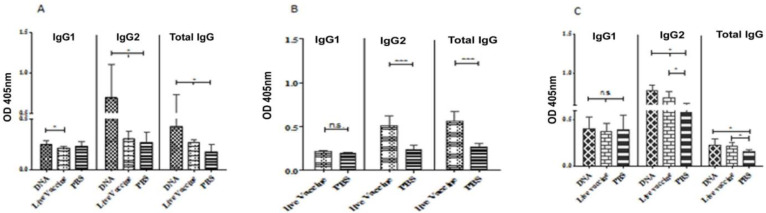
Analysis of the specific humoral response induced in hamsters after immunization with DNA/DNA and Live/Live modalities, before and after challenge. Hamsters were bled and sera were obtained after vaccinations (before challenge, 9 per group) and 5 weeks after challenge (6 per group). Sera collected at before challenge was tested for Anti-rA2-rCPA-rCPB (A) and F/T *L. tarentolae* A2-CPA-CPB^-CTE^ (B). Sera collected after challenge was tested for anti F/T *L. infantum* (C) antibodies by an isotype-specific ELISA. The results are shown as mean±SD. Student’s t test and ANOVA tests were used for statistical analysis. * *P*<0.05, **P<0.01, ***P<0.001. Optical density (OD)

Compared to infected hamsters, the level of anti rA2-rCPA-rCPB IgG2 increased in vaccinated G1 (Fig. 3A). The level of anti rA2-rCPA-rCPB IgG2 in the G1 was 0.693±0.412 before challenge that was significantly higher than other groups (Fig. 3A). Moreover, the levels of anti *L. infantum* lysate IgG2 were significantly higher in groups G1 and G2 (0.784±0.068 and 0.686±0.080, respectively) five WAC, compared to the control group (G3, 0.580±0.037) (Fig. 3C). Furthermore, anti *L. tarentolae*-A2-CPA-CPB^-CTE^ lysate IgG2 level in group 2, which was immunized with Live/Live modality, was two folds higher than that of group 3 (PBS, Control) 6 weeks after immunization and prior to parasite challenge (*P*<0.001) (Fig. 3B). Total IgG content and IgG2 levels changed with similar trends after challenge, whereas there are no significant differences in levels of IgG1 among all groups (Fig. 3A, B and C).

## Discussion

There is an urgent need to develop a vaccine against *L. infantum*, due to its increasing incidence, drug resistance and morality rate. DNA vaccine have the ability to induce cellular immune response, however, the level of vaccine induced immunity might be insufficient due to poor transfection efficiency and gene expression. Therefore, adjuvants and/or delivery systems could enhance their application. Another promising vaccination strategy is the use of nonpathogenic *L. tarentolae* harboring immunogenic antigens. This parasitic vector is able to increase antigen presentation and elicit effective immune responses without the risk of disease progression in human.

In this study, we reported a model that mimics the natural course of VL infection in hamsters by the intracardiac (i.c.) inoculation of 10^7^ *L. infantum* promastigotes. It has been demonstrated that exogamic hamsters represent a proper animal models for the discovery of antigens with potential use in the control of canine visceral leishmaniasis (33). This model allows us to study progressive VL due to *L. donovani* or *L. infantum*, which leads to hepatosplenomegaly, hypoalbuminemia and pancytopenia (34, 35).

In our previous studies, we observed that both pcDNA-A2-CPA-CPB^-CTE^ and recombinant *L. tarentolae*-A2-CPA-CPB^-CTE^ vaccinated BALB/c mice were protective against *L. infantum* suggesting that SLN is more suitable adjuvant, antigen delivery system and effective compared with electroporation (30, 36). Besides, using recombinant *L. tarentolae*-A2-CPACPB^-CTE^ as a live vaccine suggested that this novel vector might be applied to produce more strong and effective vaccines. An important advantage of this system is that it better mimics the natural development of immunity, for instance by providing a few number of live parasites necessary for maintaining stable immunity (36).

It is important to emphasize for further possible human use, it is necessary to delete the antibiotic resistance gene from the recombinant parasite. This is common concern for all recombinant live vaccine.

In the present study, these two vaccine regimens were examined in hamsters as an animal model comparable with human. It seems that cSLN formulation could improve immune responses at the first step of immunization and challenge, then electroporation surpassed at the next steps. Therefore, DNA-cSLN/DNA-electroporate was chosen as a prime-boost vaccination regime in hamster experiments. Live/Live prime-boost vaccination regime was tested in hamsters as well.

Measurement of hepatic and splenic parasite loads and antibody levels at various intervals after *L. infantum* infection revealed an interesting picture of disease progression and immunological response in adult hamsters.

The hamster body weight did not change (data not shown), since disease duration was set just till 56 days post challenge, whereas significant parasite inhibition was noticed in hamsters immunized with either DNA/DNA or Live/Live strategies at 56 days after challenge both in liver and spleen. The parasite loads in both visceral organs were controlled in vaccinated groups (G1 and G2) reaching a negligible level by day 56 post challenge compared to the control group (G3), which indicates the high efficacy of the vaccination strategy. Moreover, the much slighter increase in liver and spleen parasite burden between the fourth and eighth weeks after challenge in the vaccinated groups compared to the control group indicates the ability of these vaccines to control the disease.

Apart from decreased cellular responses, VL causes the production of high levels of antibodies, which have been observed prior to detection of parasite-specific T-cell response (37). Unlike in mice, where IL-4 and IL-12 direct IgG subclass switching of IgG1 and IgG2a respectively, such distinct IgG classes remain obscure in hamsters also IgG2 titer varied significantly between DNA-immunized and infected hamsters while IgG1 titer remained relatively constant among vaccinated hamster groups in comparison with respective infected control. In this study, the anti-rA2-CPA-CPB IgG2 antibody response became detectable after a double intra dermal injection of pcDNA-rA2-CPA-CPB^-CTE^ (group 1) 6 weeks after immunization and prior to parasite challenge. At the same time, the anti-*L. tarentolae-*A2-CPA-CPB^-CTE^ lysate IgG2 antibody response exceeded 2-fold higher in group 2, which was immunized with Live/Live modality, compared to group 3 (PBS control). Next, as early as five weeks post-infection by *L. infantum*, hamsters which received both DNA and live vaccines produced higher levels of anti-*L. infantum* lysate antibodies than the animals injected with PBS. The significant increase in the level of IgG2 only in vaccinated animals is indicative of improved cell-mediated immunity.

## Conclusion

Herein, in addition to positive results in a mouse model of infection, hamsters could be partial protected with DNA and live vaccines generated by the combination of A2-CPACPB^-CTE^ genes. Hamsters appear to be more appropriate disease models and the development of immunological tools would greatly enhance their applicability but has some drawbacks. For instance, there is no obvious way to monitor developing visceral leishmaniasis in hamsters until the very late stages of infection, when the most notable feature is a significant weight loss and the absence of clinical signs. In addition, infections often last in excess of 120 days often with no outward or measurable signs of a successful infection evident until 80–100 days post-inoculation. Furthermore, due to no intravenous route to infect hamsters, inoculation is most commonly achieved via the intracardiac route which is both technically difficult and highly hazardous for the animal (38).

As it has shown in our results, the level of IgG2 was higher than IgG1 which could act as reasonable parameter of IFN-γ production.

Furthermore, using the golden hamster model of visceral leishmaniasis is shown that parasite burden in liver and spleen and humoral immune responses could be useful tools for estimating the activity of new vaccine formulations*.*

## Ethical considerations

Ethical issues (Including plagiarism, informed consent, misconduct, data fabrication and/or falsification, double publication and/or submission, redundancy, etc.) have been completely observed by the authors.
